# Coral Reef Habitat Response to Climate Change Scenarios

**DOI:** 10.1371/journal.pone.0082404

**Published:** 2013-12-05

**Authors:** Lauren A. Freeman, Joan A. Kleypas, Arthur J. Miller

**Affiliations:** 1 Scripps Institution of Oceanography, La Jolla, California, United States of America; 2 National Center for Atmospheric Research, Boulder, Colorado, United States of America; National Oceanic and Atmospheric Administration/National Marine Fisheries Service/Southwest Fisheries Science Center, United States of America

## Abstract

Coral reef ecosystems are threatened by both climate change and direct anthropogenic stress. Climate change will alter the physico-chemical environment that reefs currently occupy, leaving only limited regions that are conducive to reef habitation. Identifying these regions early may aid conservation efforts and inform decisions to transplant particular coral species or groups. Here a species distribution model (Maxent) is used to describe habitat suitable for coral reef growth. Two climate change scenarios (RCP4.5, RCP8.5) from the National Center for Atmospheric Research’s Community Earth System Model were used with Maxent to determine environmental suitability for corals (order Scleractinia). Environmental input variables best at representing the limits of suitable reef growth regions were isolated using a principal component analysis. Climate-driven changes in suitable habitat depend strongly on the unique region of reefs used to train Maxent. Increased global habitat loss was predicted in both climate projections through the 21^st^ century. A maximum habitat loss of 43% by 2100 was predicted in RCP4.5 and 82% in RCP8.5. When the model is trained solely with environmental data from the Caribbean/Atlantic, 83% of global habitat was lost by 2100 for RCP4.5 and 88% was lost for RCP8.5. Similarly, global runs trained only with Pacific Ocean reefs estimated that 60% of suitable habitat would be lost by 2100 in RCP4.5 and 90% in RCP8.5. When Maxent was trained solely with Indian Ocean reefs, suitable habitat worldwide *increased* by 38% in RCP4.5 by 2100 and 28% in RCP8.5 by 2050. Global habitat loss by 2100 was just 10% for RCP8.5. This projection suggests that shallow tropical sites in the Indian Ocean basin experience conditions today that are most similar to future projections of worldwide conditions. Indian Ocean reefs may thus be ideal candidate regions from which to select the best strands of coral for potential re-seeding efforts.

## Introduction

Anthropogenic climate change will alter many physical and chemical characteristics that comprise the niches of marine species and ecosystem habitats. Changes in these physico-chemical conditions are already leading to shifts in the habitat ranges of some marine species [1], and extinction rates of marine species are expected to increase [2,3]. The environmental conditions in which many ecosystems have evolved are shifting geographically. Consequently, suitable habitat spaces for these ecosystems are being ‘re-mapped’ in accordance with changes in multiple environmental variables. Projecting the geographic distribution of these future marine habitat areas is made difficult by our incomplete knowledge of not only the physico-chemical limits of marine habitats, but the biological and ecological limits of the species that occupy these habitats (e.g., the role of species interactions in defining ecosystems). Habitat niche models, which predict suitable habitat envelopes for a given species or group of organisms, are a good first estimate of habitat requirements. Habitat niche models can thus be used to estimate the future geographic range of appropriate habitat areas in climate change scenarios.

As climate change alters oceanographic conditions, the geographic range of ocean habitat suitable for the growth of coral reef ecosystems is shifting. Corals of the order Scleractinia provide the foundation of shallow-water coral reef ecosystems. Scleractinia corals secrete calcium carbonate skeletons, which accumulate as reef structures that in turn support highly biodiverse communities. All shallow-water, tropical coral reefs are defined by the same broad ecological functional groups, but they develop across a wide array of ocean environments [4]. The mean environmental conditions in which reefs are found differ across the three tropical ocean basins (Caribbean/Atlantic, Pacific, and Indian), and there is further variation within each basin [4]. While individual coral colonies are acclimatized to the conditions of their own unique location, each region will experience a different combination of environmental shifts associated with climate change.

Increasing sea surface temperatures and extreme temperature excursions are considered a major threat to coral reef ecosystems primarily because they have been shown to be the major factor behind the recent global increase in coral bleaching [5,6]. Ocean acidification has also been shown to affect coral colonies in multiple ways, including reducing the ability of coral polyps to secrete calcium carbonate skeletons, and reducing the integrity of the reef structure [7]. The geographical limits of shallow-water coral reef ecosystems are also defined by salinity, light availability as photosynthetically active radiation (PAR), water current speed, [4,8], and other variables that are more difficult to quantify such as species interactions and connectivity.

This article considers the geographical change in physico-chemical environments appropriate for coral reef ecosystems, as projected by the National Climate and Atmospheric Research (NCAR) center’s Community Earth System Model Version 1 (CESM1) in a suite of climate change scenarios. Suitable reef habitat was defined using a niche model, Maxent [9], through analysis of the environmental envelope in which coral reefs exist in the present-day. In combining model outputs and suitable environmental parameters as defined by Maxent, projections are made regarding how these physico-chemical changes may influence the spatial distribution of suitable habitat. The environmental variables projected by CESM1 include key limiting variables to reef development. The results yield a range of possible future states regarding the world-wide spatial distribution of coral reef ecosystems as they experience climate change. Combining CESM1 and Maxent, two state-of-the-art tools, provides a projection of coral reef ecosystem survivability in up-to-date climate change scenarios. While a number of caveats are raised due to the use of species distribution models (SDMs) and similar tools in projecting future habitats [10,11], these projections provide some insight toward actions that could help guide coral reef ecosystem preservation. For example, resources may be better utilized by focusing conservation efforts on those areas where suitable reef habitats are likely to persist under future climate conditions. 

## Materials and Methods

Potential coral reef habitat was modeled using Maxent, a maximum entropy niche model that performs well with presence-only data for species or communities [9]. Maxent is based on deterministic algorithms that converge to the optimum (maximum entropy) probability distribution of habitat suitability across a spatial domain. Environmental inputs for the model were based on climate simulation outputs for the present day from CESM1. Present-day coral reef distributions, obtained from ReefBase [12], were used to train Maxent.

### 2.1: Climate model data

CESM1 is a global atmosphere-ocean, fully coupled climate model that includes global carbon cycling. CESM1 is built on the Community Climate System Model version 4 (CCSM4), with a nominal horizontal resolution of 1° by 1° which is enhanced in the tropics and at high latitudes [13]. The ocean component of the global carbon cycle includes the Biogeochemical Element Cycle (BEC) model of Moore et al. [14], which includes four nutrients (N, P, Si, Fe), three phytoplankton functional types (diatoms, pico/nano-phytoplankton, and diazotrophs), and one zooplankton class. The model determines the complete suite of carbonate system components (pH, pCO_2_, HCO_3_^-^, CO_3_^2-^, and alkalinity), which are then used to calculate calcium carbonate (aragonite) saturation state [15]. 

Model data were extracted from the CESM1 BEC 20^th^ century run (1985–2005) and two 21^st^ Century scenario runs described by unique representative carbon pathways (RCPs): RCP4.5 (2005–2100) and RCP8.5 (2005–2100). The scenarios yield differences of approximately 2°C in mean global air temperature. Ocean data were retrieved only from the surface layer (the upper 10 m), as most shallow-water coral growth occurs in the upper littoral zone (shallower than 30 m). The absolute increase in mean sea surface temperature for the domain considered is 0.73° C for RCP4.5 and 2.10° C for RCP8.5. The variables extracted from CESM1 included sea surface temperature, sea surface salinity, phosphate concentration at the sea surface (PO4), average light availability in the surface layer as PAR, surface horizontal current velocities, and carbonate system parameters. Since concentrations of the nutrients nitrate, phosphate, and silicate are strongly correlated in seawater [16], only phosphate was retained.

Monthly output from the CESM1 biogeochemistry model simulations of the 20^th^ century (for training Maxent), and the two 21^st^ century simulations RCP4.5 and RCP8.5, were interpolated to a grid with 1° by 1° resolution. The monthly data were averaged over 20-year time periods, from 1985–2005 for the 20^th^ century model and as overlapping time periods in the 21^st^ century runs (2011–2030, 2021–2040, etc.) until 2100. As the 21^st^ century runs begin at year 2005, data were also collected for the ten year time period 2006–2015 to calculate an average for 2010. These files were converted to Matlab® structure files for processing in Matlab® (R2011a). This process is described in more detail below in Section 2.3. 

### 2.2: Biogeographic data

Coral-reef ecosystems presently exist in limited regions of the tropical and subtropical oceans. Data regarding the specific locations of these ecosystems are available from ReefBase (www.reefbase.org). ReefBase locations are provided by the Millennium Coral Reef Mapping Project [12]. These spatial data were interpolated to the same 1° by 1° resolution grid as the CESM1 data. Ocean basins were divided by geographic boundaries into the Indian, Pacific, and Caribbean/Atlantic basins. These three sub-regions allow for separate application of the habitat niche model through training it for one particular subset of reefs restricted to the basin in which they reside, as described in section 2.4. Coral-reef ecosystems also exist in the Red Sea and Persian Gulf. The unique oceanographic conditions of these two regions would require their treatment as separate sub-regions, none of which contain a sufficient number reef locations once interpolated to a 1° by 1°grid to adequately train the niche model. Consequently these regions are not included in this analysis.

### 2.3: Determination of environmental variables

Surface current speeds were calculated from the horizontal velocity components of the CESM1 data. Aragonite saturation state (Ω_arag_) was calculated as the ratio of carbonate ion concentration and carbonate ion concentration at Ω_arag_ =1, from the CESM1 carbon-system components. The mean, maximum, minimum, and annual range for current speed, Ω_arag_, sea surface temperature, sea surface salinity, sea surface phosphate concentration, and surface PAR were obtained for each of the 20-year time periods. Averaging across a 20-year time period provided an estimate of the climatological state from CESM1. All variables are normalized prior to subsequent analyses.

From temperature (SST), further calculations were performed to estimate cumulative thermal stress (CTS), as shown in (1). CTS is similar to the degree heating week measure (e.g. 17), and is calculated as the temporal accumulation of the excess monthly SST when SST exceeded the mean monthly maximum (MMM) plus two standard deviations of mean monthly maximum for each grid cell [18]. Using two standard deviations (as opposed to one degree Celsius, used to calculate degree heating weeks) is more effective for the broad geographic area considered as well as for monthly data (versus weekly). Equation (1) describes the method by which cumulative thermal stress was estimated:

CTS(x,y,t)=SST(x,y,t)−[MMM(x,y)+2σMMM] (1)

where *x* represents latitude, *y* represents longitude, *t* represents time, *SST* represents sea surface temperature, and *σ*_*MMM*_ represents the standard deviation of the MMM. MMM data were calculated from the 1985–2005 20^th^ century run, and CTS was then calculated and summed over each 20-year simulation period. These data are not a direct analog to degree heating weeks, but rather represent the overall thermal stress for each 20-year time period considered in this study. 

**Figure 1:**
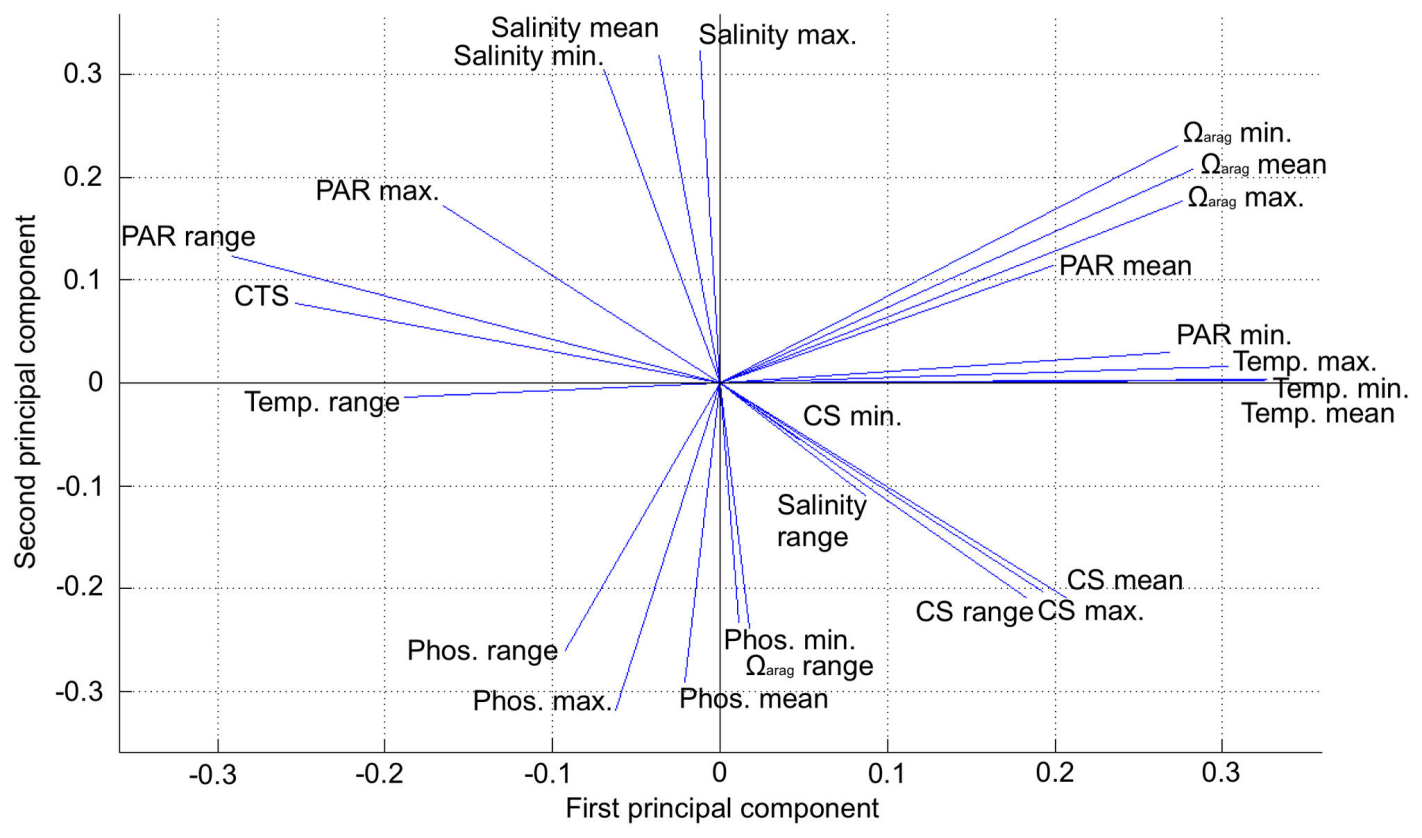
The 25 environmental variables initally considered in this study as projected into principle component space. This projection shows the correlation between variables, as equivalent to the cosine of the angle between vectors. Tightly clustered vectors are used as groups from which final variables were selected to minimize aliasing in subsequent analyses. Each vector group is detailed in List S1. CS = Current Speed; PAR = Photosynthetically Available Radiation.

To reduce aliasing between variables and to improve the performance of Maxent, highly correlated variables were eliminated using principle component analysis (PCA) for three time periods in the 21^st^ century (2011–2030, 2041–2060, and 2081–2100). Each of these runs yielded two dominant (25-element) structure functions that combined explain at least 60% of the total variance among the spatial locations, and each generated similar structure functions that revealed correlations among the variables when plotted along perpendicular axes (Figure 1). Six groups of highly correlated variables consistently arose in the PCA for each time period and scenario. Table S1 shows that similar results prevail when computing Spearman rank correlations among variables directly. From each group of correlated variables, only one was chosen for use in final calculations based on the following two criteria: the known importance of the variable to coral-reef ecosystem habitat state, and whether the variable was likely to change appreciably in future climate scenarios. The resulting list of variables included CTS, phosphate maximum, current speed maximum, salinity minimum, PAR minimum, and Ω_arag_ minimum. 

**CTS**: (strongly correlated with PAR annual range) provides an indication of the duration and magnitude of temperature excursions that exceed the tolerance of coral organisms. Temperature stress is strongly correlated with coral bleaching [17], which often leads to partial or complete mortality of individual coral colonies. CTS is expected to increase in most reef locations as the effects of climate change intensify.

**Phosphate maximum**: (strongly correlated with Ω_arag_ annual range; and PO4 minimum, annual range, and mean) is also considered representative of nitrate and silicate levels in this study. Nutrient enrichment is often associated with coral-reef ecosystem degradation as it can lead to a shift in community structure towards one dominated by fleshy macroalgae [19]. Climate change may cause a shift in nutrient patterns in the future through changes in circulation and increased ocean stratification.

**Current speed maximum**: (strongly correlated with PAR maximum, annual range of salinity; current speed minimum, mean and annual range) is an indication of the hydrodynamic energy in the surrounding environment. Currents are important to coral-reef ecosystems because they provide a well-oxygenated environment that enhances food availability and provides flushing of reef waters. High current speeds have been correlated with reduced heat stress and thus less coral bleaching and mortality in several previous studies [20-22]. 

**Salinity minimum**: (strongly correlated with maximum and mean salinity). Most reef building coral organisms are intolerant of salinities less than 25 parts per thousand (ppt), [23], and no coral reefs are found where the minimum monthly salinity is less than 23 ppt [8]. Climate-related changes in rainfall patterns could affect salinity regimes in the future, particularly in coastal regions.

**Ω_arag_ minimum**: (strongly correlated with mean PAR; Ω_arag_ mean and maximum) is a measure of the degree of aragonite saturation in seawater. Both the skeletal growth rate in individual coral colonies and the rate of increase in the quantity of reef-building framework have been correlated with Ω_arag_ [24]. Because ocean acidification is causing significant decreases in Ω_arag_ , regions of high Ω_arag_ are contracting in area, mostly equatorward [25,26]. Once atmospheric CO_2_ levels double in comparison to preindustrial levels (estimated occur at approximately 2050 in RCP 8.5), the combined effects of coral bleaching and decreased Ω_arag_ will decrease net calcification on reefs to the point that most will shift from net reef-building to net dissolution [26,27].

**PAR minimum**: (strongly correlated with temperature mean, maximum, annual range, and minimum) represents the amount of radiation available to organisms for photosynthesis. As coral polyps house photosynthesizing symbiotic dinoflagellates, PAR is critical for their survival. Minimum PAR levels can be limiting to suitable reef habitat area, particularly at high latitudes where persistently low PAR can limit coral structures to very shallow regions [8]. Climate change may affect the distribution and opaqueness of clouds [28], which in turn could affect PAR in reef habitats. 

### 2.4: Bioclimatic envelope modeling

Maxent is a maximum entropy niche model that uses environmental variables (also termed “layers”) combined with species presence data to determine the likelihood of suitable habitat for that species at each grid cell within a geographic domain [9,29]. Maxent has been successfully used to identify suitable habitats for endangered species (e.g. 30), to map potential habitats for cold water coral reefs (e.g. 31-33), and to understand modern environmental limits to shallow-water coral reef development in the tropics [34,35]. Couce et al. [35] determined that Maxent performed well in projections of the present-day distribution of shallow-water coral reefs. Their results showed that temperature-related variables were the most important in accurately modeling present-day reef distribution, followed by Ω_arag_, nutrients, and light [35]. Here, climate model projections of a similar suite of variables are used as layers in the Maxent model. These extend the use of Maxent from considering only present-day spatial distributions of coral reefs to exploring how the envelope of suitable coral reef habitat (the “bioclimatic envelope” following the terminology of [36]) might change in the future. The data ‘jackknifing’ analysis in Maxent confirmed our PCA-based choice of input variables as the most relevant to predicting the suitability of a habitat for coral reef presence. 

Maxent version 3.3.3 (http://www.cs.princeton.edu/~schapire/maxent/) was first trained using the world-wide distribution of coral reefs and the six selected environmental parameters from the CESM1 model output for the time period 1985–2005. The model was trained using 75% of reef locations, selected randomly, and testing of model performance was implemented by predicting the remaining 25% of reef locations. For the first case considering all reefs, 894 locations were used for training and 298 additional locations were used for testing. For the Indian Ocean case, 146 sites were used for training and 48 for testing; for the Pacific Ocean case, 569 sites were used for training and 189 for testing; and for the Caribbean case 135 locations were used for training and 45 additional sites were used for testing. For each of the 72 sets of Maxent runs, the mean AUC score (an indicator of model performanc) was greater than 0.8 with the ‘Clamping’ function enabled. 

To project how the spatial distribution of coral reef habitat may change in the future, Maxent was run with the 21^st^ Century CESM1 output for the overlapping 20-year time periods in both RCP scenarios. Projections were first run based on training with the global distribution of coral reefs. Projections were also run based on training restricted to three separate coral reef domains corresponding to the Caribbean/Atlantic, Pacific, and Indian Ocean basins. For these three cases, the Maxent model was trained using a subset of coral reef distribution that only included regions where corals are found within each ocean basin. These basin-specific projections were run to examine how the suitable environmental area in which coral reefs specific to each domain reside would shift geographically both within and outside each domain during the specified climate change scenarios. Predictions outside of the ‘home’ domain enabled Maxent to estimate where coral reef habitat, as represented in each basin, would theoretically exist within other ocean basins. 

These projections of suitable habitat areas for coral reef growth in the future do not take into account bathymetry. That is, suitable habitat illustrates regions where the phsyico-chemical conditions are adequate for coral reef growth, but in reality reef growth within these habitats will be restricted to water depths of approximately 30 m or less.

## Results

**Figure 2:**
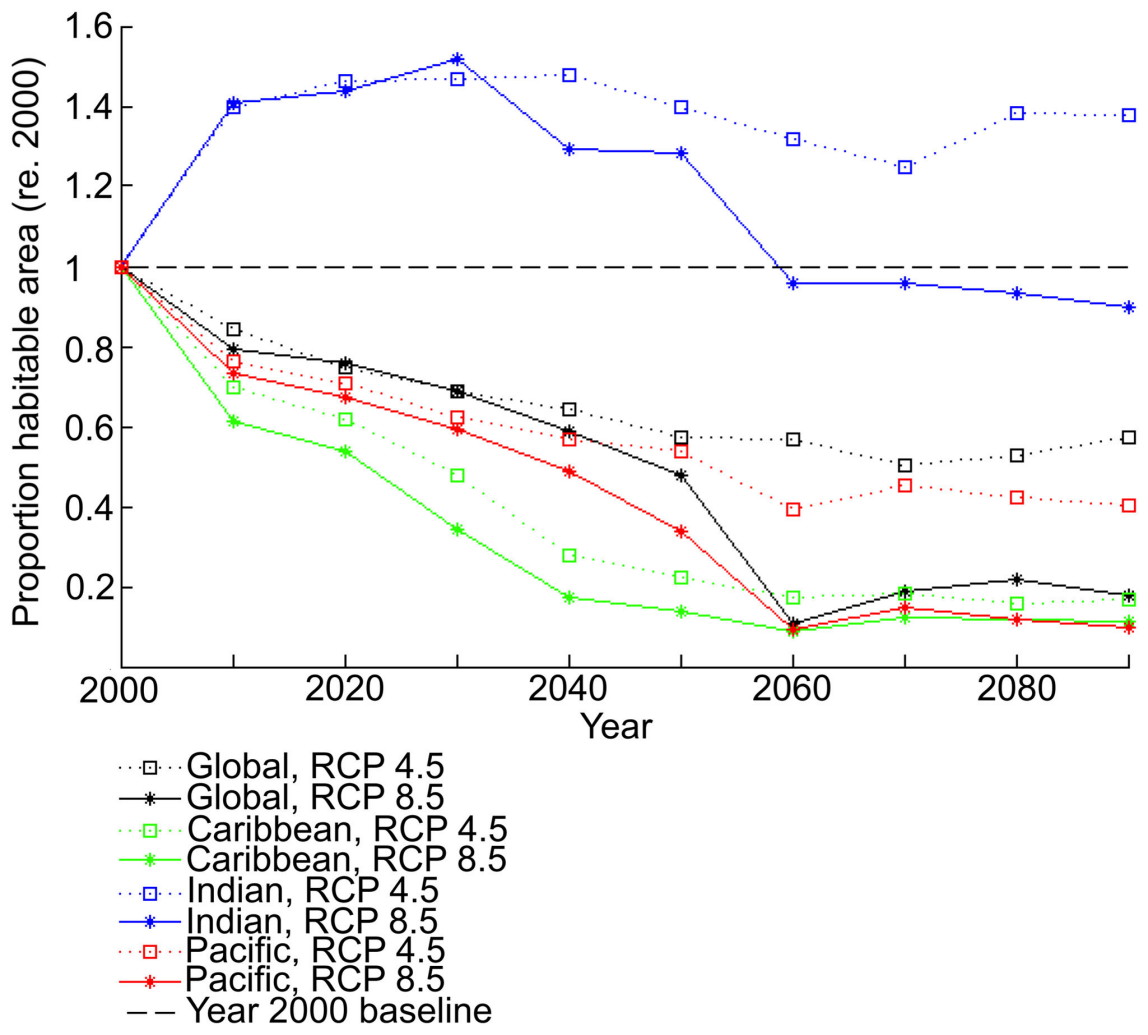
Time series showing the estimated change in habitable area over time, as compared to what was available in the 20^th^ century. Average habitat suitability worldwide was calculated from NCAR CESM1 data for the year 2000, then for future climate projections. Global model runs considering all reefs are marked in black, runs considering only Caribbean reefs to train Maxent are marked in green, runs considering only Indian reefs to train Maxent in blue, and runs using only Pacific reefs in red. The less extreme RCP4.5 projection is indicated by squares and dotted lines, while the more extreme RCP8.5 is indicated by stars and solid lines. No change from 20^th^ century habitable area (2000) is represented by the dashed black line.

Compared with Maxent’s prediction of the 1985–2005 distribution of reefs, projected coral-reef habitat declined with time in both the RCP4.5 and RCP8.5 climate scenarios (Figure 2). Percent change in habitat is calculated as the summation of habitat suitability from the RCP scenarios divided by the summation of habitat suitability in the training run. By the year 2100, the area of suitable habitat over the global domain was reduced by 43% in the RCP4.5 scenario, and by 82% in the RCP8.5 scenario (Figure 2). 

When Maxent was trained separately for each ocean region using the basin-specific physico-chemical preferences associated with the presence of coral reefs, the resulting projections of suitable habitat area for both the Pacific and Caribbean/Atlantic reefs were similar to when the global distribution of reefs was used (Figure 2). The envelope of Pacific coral reef habitat was reduced globally by 90% by 2100 in the RCP8.5 scenario, and the global envelope of habitat suitable for Caribbean/Atlantic coral reefs was reduced by 89%. However, when Maxent was trained with the spatial distribution and physico-chemical data for Indian Ocean reefs, the global envelope of suitable habitat area for Indian Ocean coral reefs increased by 38% in RCP4.5 by 2100 and decreased by only 10% in RCP8.5 by 2100 (Figure 2).

The global runs, as well as those considering only Indian reefs and only Pacific reefs, all experience a precipitous drop in percent of suitable habitat from 2050 to 2060 in the RCP8.5 scenario only. This is mainly a result of a dramatic change in Ω_arag_ minimum in the climatological variables calculated from CESM1 (20 year averages) from the 2050 mean to 2060 mean. The average for the entire study area is 2.48 in 2050, 1.87 in 2060, and recovers to 2.15 in 2070. Suitable habitat in the Caribbean/Atlantic had already decreased by over 70% in 2050 and does not show the same drop. 

**Figure 3:**
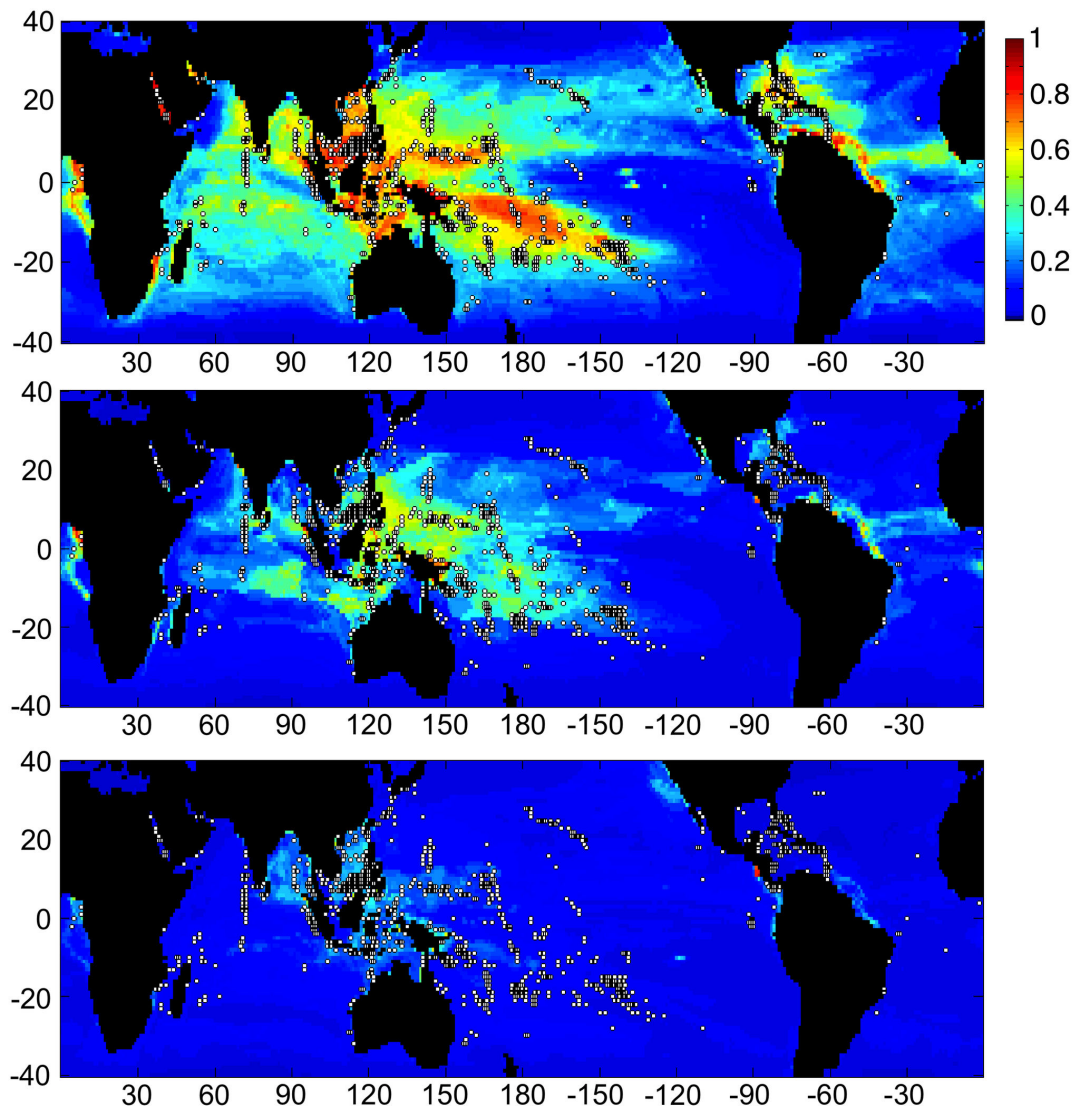
Three charts showing habitat suitability estimates when Maxent was trained using the current location of coral reefs worldwide (white dots), and based on CESM1 model output for the RCP8.5 scenario. Color scale indicates the probability that conditions are suitable for reefs: red = high probability, green = average probability (typical conditions for present-day reefs), blue = low probability. Horizontal axes indicates longitude, while vertical axes indicate latitude from 40° north to 40° south. Charts from top to bottom present results from the training run from 2000, estimates for 2050, and estimates from 2100, respectively. The 2100 RCP4.5 projection is similar to the 2050 RCP8.5 projection shown here.

**Figure 4:**
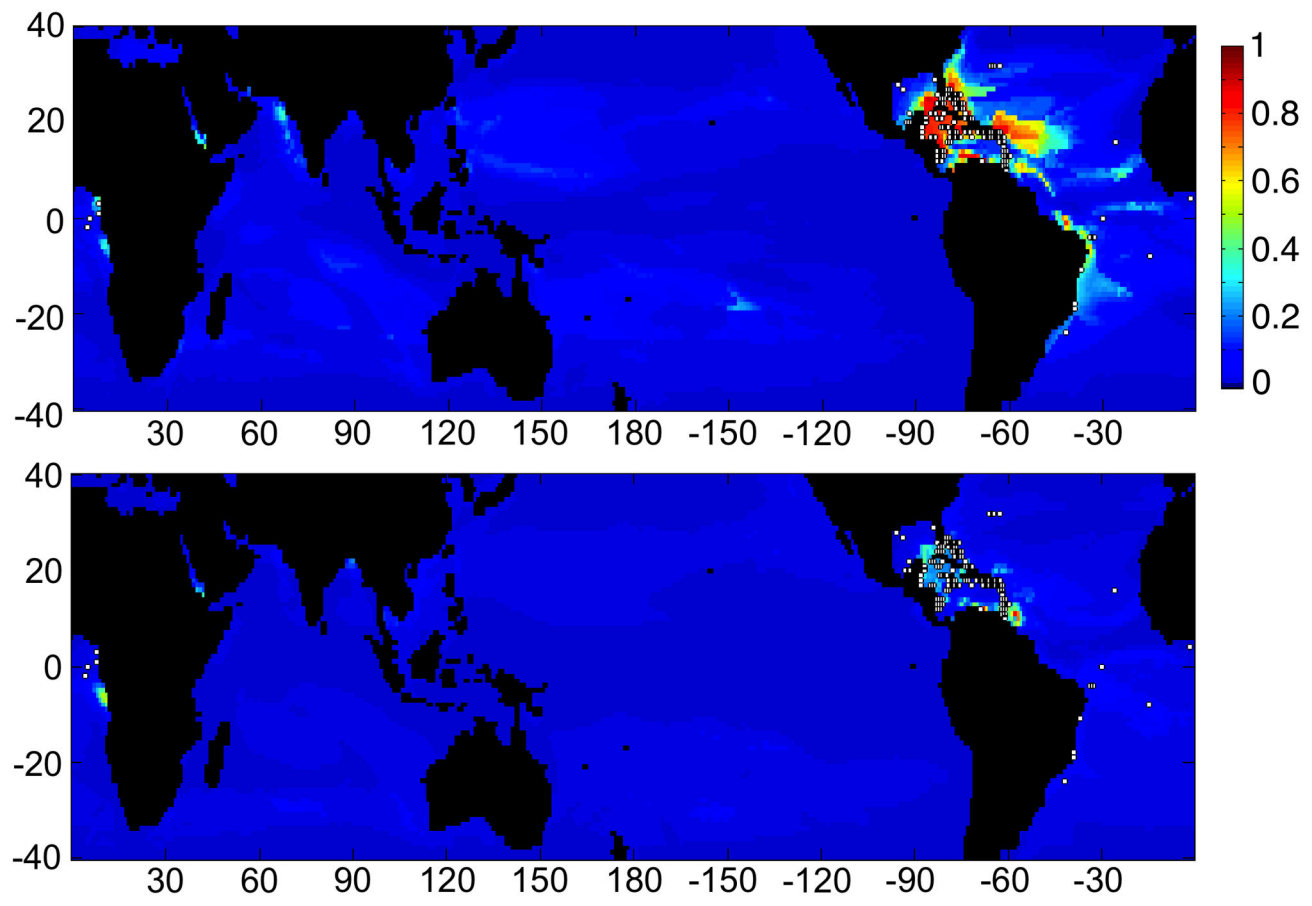
Two charts showing habitat suitability estimates when Maxent was trained using the current location of reefs within the Caribbean (white dots), and based on CESM1 model output for the RCP4.5 scenario. Charts from top to bottom present results from training based on current climate CESM1 data from 1985–2005, and estimates for conditions in 2050, respectively. Color scale and axes are identical to Figure 3. Note that present habitat suitable for Caribbean/Atlantic reefs is shown globally. These are model results and do not consider the ability of specific corals to migrate between basins, as no coral species are shared between the Caribbean and the Indo-Pacific. Estimates for 2100 conditions, as well as those for RCP8.5 for 2050 and later revealed almost no suitable habitat for Caribbean reefs anywhere on earth.

**Figure 5:**
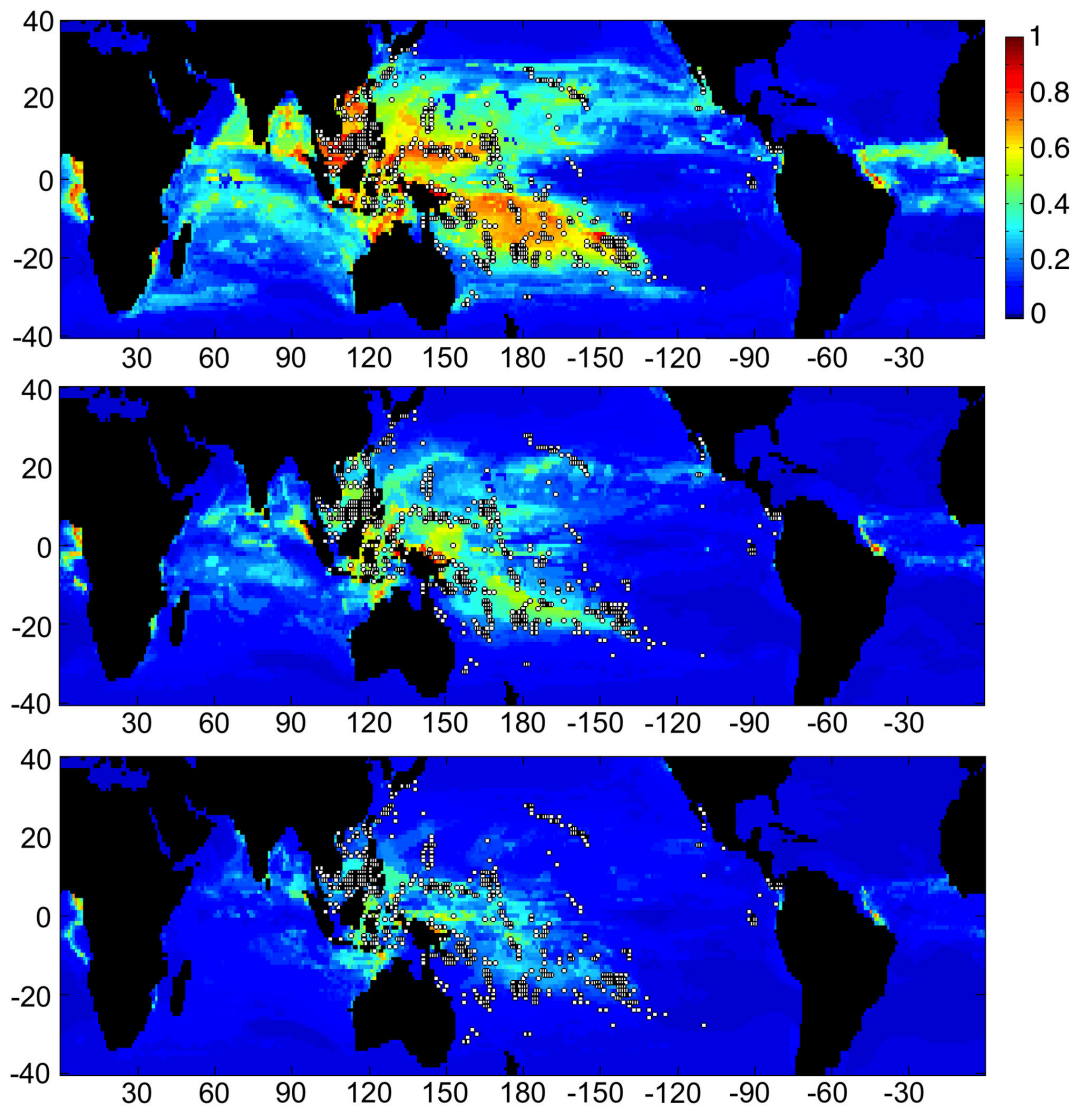
Three charts showing habitat suitability estimates when Maxent was trained using the current locations of Pacific Ocean coral reefs (white dots), and based on CESM1 model output for 2050. Color scale and axes are identical to Figure 3. Charts from top to bottom present results from training based on current climate CESM1 data from 1985–2005, 2050 conditions in RCP4.5, and 2050 conditions in RCP8.5, respectively. 2100 conditions in RCP4.5 are very similar to 2050 conditions for RCP8.5.

**Figure 6:**
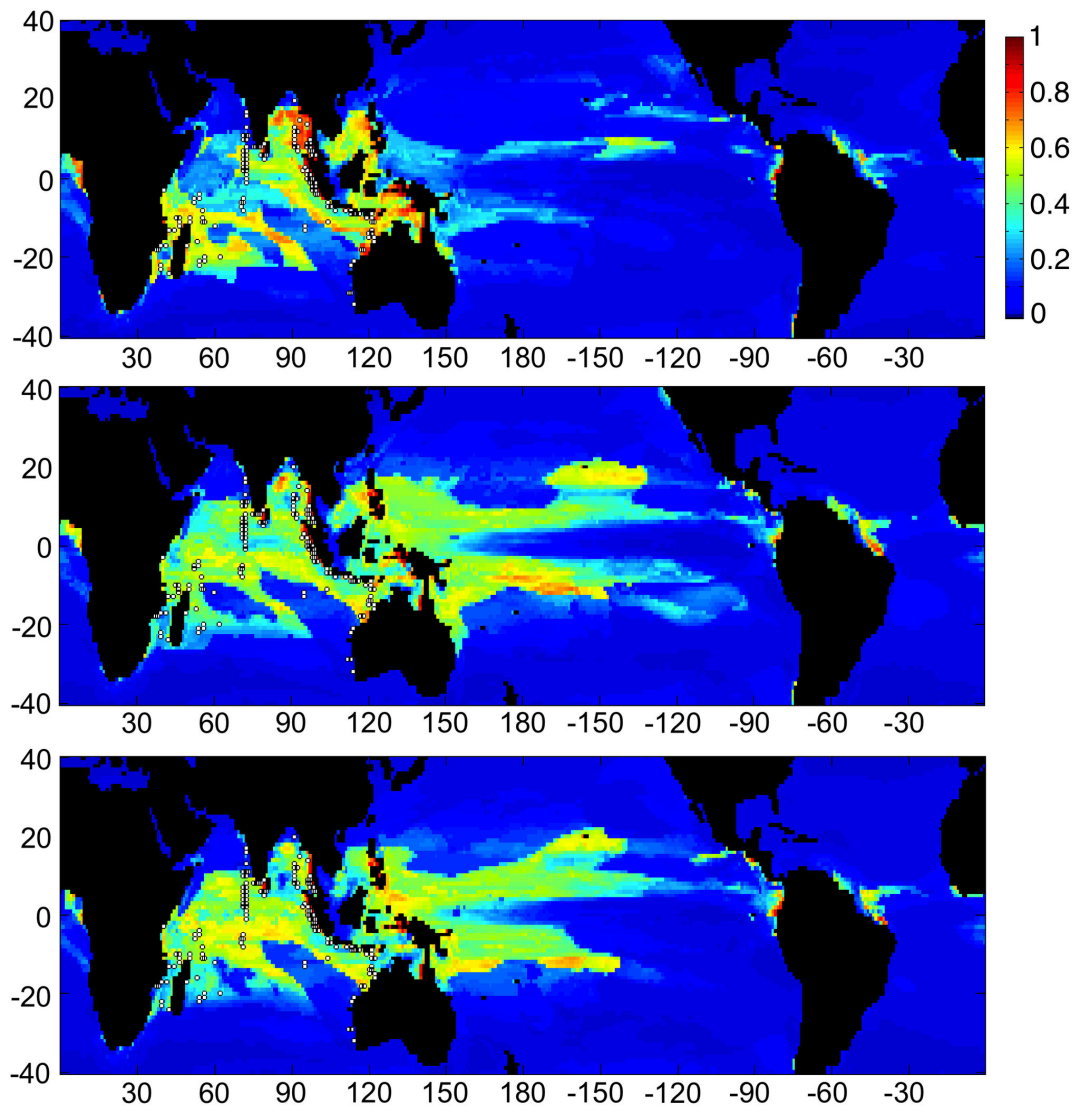
Three charts showing habitat suitability estimates when Maxent was trained using the current locations of Indian Ocean coral reefs (white dots), and based on CESM1 model output for 2050. Color scale and axes are identical to Figure 3. Charts from top to bottom present results from training based on current climate CESM1 data from 1985–2005, 2050 conditions in RCP4.5, and 2050 conditions in RCP8.5, respectively.

Spatial distributions of the relationship between physico-chemical variables and the existence of present-day of coral reefs indicate marked differences in the “habitat space” occupied by reefs from different basins. In addition, model projections suggest markedly different basin-specific shifts in those habitats (Figures 3,4,5,6). Based on the six environmental parameters used here, very little habitable space for Caribbean/Atlantic coral reefs exists today outside of their present geographic domain (Figure 4). These models estimate that the possibility of habitat expansion in the case of Caribbean/Atlantic corals is almost eliminated by the middle of this century, even in the RCP4.5 scenario. 

Under both climate change scenarios, the region of suitable habitat for Pacific reefs is reduced in a relatively uniform manner across all ocean regions. Apart from the Pacific, some suitable habitat was identified in both the Indian and the Atlantic Oceans but not in the Caribbean (Figure 5). Regions of suitable habitat were identified for Indian Ocean reefs in both the Pacific and Atlantic Oceans but not in the Caribbean (Figure 6). Under both climate-change scenarios, the area of suitable habitat for Indian Ocean reefs increases in the Pacific Ocean and to a lesser degree in the Atlantic. Patterns of habitat suitability shown in Figures 3-6 and the trends shown in Figure 2 were persistent through many iterations of Maxent using various model parameters and settings. In addition, results remained stable when the combination of input variables used to train Maxent were changed to assess the sensitivity of the model. 

## Discussion

These bioclimate modeling results indicate that climate change and ocean acidification will impact the distribution of suitable coral reef habitat in the future, for both the RCP4.5 and the RCP8.5 scenarios. Globally the envelope of oceanographic conditions favorable to coral reef development will decrease by 43% and 82% by the year 2100 for the RCP4.5 and RCP8.5 scenarios, respectively. The decreases in actual reef habitat will further depend, in part, on the existence of shallow substrate within the envelope. 

The global runs also identify where potential new coral habitats may emerge in the future. For example, rising sea surface temperatures are expected to shift the suitable habitat envelope poleward in both hemispheres, and some coral species have indeed begun to colonize at higher latitudes [37,38]. The results presented here, however, do not suggest a significant shift of coral reef habitat into new regions (Figure 3), indicating that other variables limit this expansion. Rather, the bioclimatic envelope suitable for coral reefs is predicted here to decline in all regions indicating that other variables may limit this expansion. Light availability (PAR) and Ω_arag_ have both been shown to limit coral reef development at high latitudes [8,25] and declining Ω_arag_ has been associated with a decreased ability of coral communities to construct coral reefs [25,39]. One previous study found Ω_arag_ to shift habitat to ‘marginal’ by the mid- to late-21^st^ century, but those results also indicate that regions of habitat within the margins of present-day coral reef habitation persist for at least the next 100 years [25]. 

The bioclimatic envelopes amenable to coral reef growth are defined slightly differently for each ocean basin. The changes in the projected envelopes of suitable habitat for Pacific Ocean reefs are similar to those for the global projections, while those for Indian Ocean reefs are maintained across many regions, in spite of the significant changes in modeled ocean conditions throughout the 21^st^ century. The projected envelopes of suitable habitat for Caribbean/Atlantic reefs, however, nearly disappear under both climate change scenarios. This striking difference for Caribbean/Atlantic reefs remained robust across multiple iterations using Maxent, and persisted even when removing each of the six variables individually from training. Thus the susceptibility of Caribbean/Atlantic reefs to future conditions does not appear to be an artifact of a single variable skewing the results. The modeled vulnerability of reefs in this region may simply reflect the smaller number of reef locations in the Caribbean/Atlantic used to train Maxent, which may narrow the range of environmental conditions presently experienced by reefs across the domain. The difference may also be due to the unique physico-chemical environment of the Caribbean. Regardless of the cause, these projections suggest very different fates for coral reefs that presently exist in the three major ocean basins.

In both the global and basin-scale projections, certain regions stand out for their retention of suitable reef habitat under future climate scenarios through the year 2050 and beyond. These include mostly equatorial regions in the northern Indian Ocean, the Coral Triangle region, French Polynesia, and the northeast Brazilian shelf east of the Amazon River. These regions are where the CESM1 projections estimate suitable temperatures, Ω_arag_, light, nutrients and current speeds. Among these variables, projected temperature and Ω_arag_ change markedly in the future, so that Maxent’s projection of future reef habitat strongly reflects the net effect of 1) a poleward shift of suitable habitat due to increasing temperature, and 2) a shift of suitable habitat toward the equator due to decreasing Ω_arag_. 

Our work is in line with previous predictions of coral reef decline in the 21^st^ century. One previous study argues that coral reefs will face serious decline unless atmospheric CO_2_ is limited to 350 ppm [40]. Another study based solely on sea surface temperatures in global climate models indicated that approximately two-thirds of coral reefs worldwide faced degradation by the end of the 21^st^ century under the RCP4.5 scenario [41]. Our results which consider temperature as well as salinity, nutrient levels, current speeds, light availability, and Ω_arag_ similarly predict a loss of at least 43% of habitable area worldwide under the RCP4.5 scenario.

The results also indicate which basins presently include reef habitats that are “best conditioned” to spread to new regions. Present-day coral reefs in the Indian Ocean basin experience conditions that are most similar to future climate projections in the Indian as well as the Pacific and Atlantic Oceans (Figure 6). Another previous study found that Arabian and Persian Gulf reefs are amongst the most heat-adapted in the world, and argue for assisted migration of these corals to the Indo-Pacific [42]. The shifting of suitable habitat space for a number of species has led some to consider species translocations or “managed relocations” – by introducing these species into new regions with suitable conditions – in order to conserve them [43-45]. A more synoptic motive for translocation, and much less studied, is to introduce a substitute for a foundation species, such as a dominant reef-building coral, with the goal of restoring or maintaining ecosystem function [44,46]. The topic is justly controversial [47] but managed relocations and introductions may become more acceptable in cases where climate change severely limits more traditional conservation strategies [45]. Should a major reef-building species decline due to factors associated with climate change, it may be advantageous to introduce an alternate reef-building species that is more suited to the new environment to maintain reef functionality. What would be the impact, for example, of introducing a species of reef-building coral endemic to the Indian Ocean to Caribbean reefs that have already seen the decline of several major reef building species [48], and which appear to be particularly vulnerable to climate related physico-chemical changes? 

**Figure 7:**
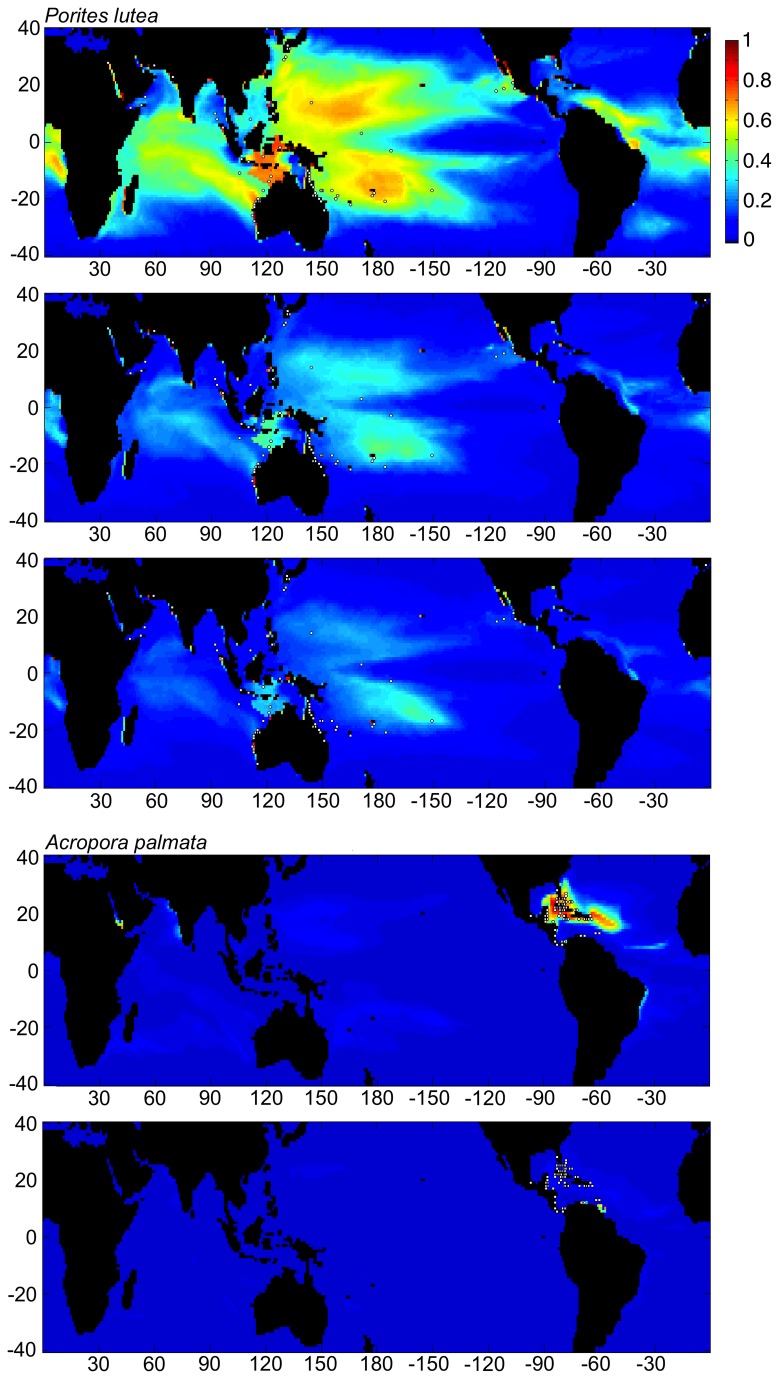
Two charts showing habitat suitability estimates when Maxent was trained using the current location of reefs within the Caribbean (white dots), and based on CESM1 model output for the RCP4.5 scenario. Charts from top to bottom present results from training based on current climate CESM1 data from 1985–2005, and estimates for conditions in 2050, respectively. Color scale and axes are identical to Figure 3. Note that present habitat suitable for Caribbean/Atlantic reefs is shown globally. These are model results and do not consider the ability of specific corals to migrate between basins, as no coral species are shared between the Caribbean and the Indo-Pacific. Estimates for 2100 conditions, as well as those for RCP8.5 for 2050 and later revealed almost no suitable habitat for Caribbean reefs anywhere on earth.

Answering the question above would require considerable further research, including a more comprehensive niche-based approach that addresses individual species and their biotic as well physical-chemical environments. Maxent and other niche distribution software packages are typically used for species distributions, although Maxent has been used in the larger sense for coral-reef ecosystem distributions [34,35], as has been done here. The ‘niche’ used in this study is the bioclimatic envelope that spatially correlates with net reef growth, rather than individual species. As a result, this envelope includes a composite of all niches that include reef-building species of coral. Under the effects of climate change, the spatial distribution of each coral species will shift individually in response to changes in their individual environmental niches. These shifts may be geographically different when compared with the composite of all reef-building species. However, some of the major reef-building coral species are widespread within their ocean basins, such as *Porites lutea* in the Pacific and *Acropora palmata* in the Caribbean/Atlantic. The responses of these two species alone were tested using Maxent, and the results were similar to the composite reef results. The main difference in both tests was a more constricted range, presumably as a result of the smaller number of locations used for model training, resulting in a more restricted suitable habitat envelope. While projections of suitable habitat area for *A. palmata* showed that suitable regions essentially disappeared in the future, there is some indication that hypothetically suitable habitat for *P. lutea* could persist in some portions of the Caribbean/Atlantic in addition to the Pacific and Indian basins through most of this century (Figure 7). 

These results should be viewed with caution because recent reviews have questioned the validity of using SDMs to project changes in habitats across time [11,49]. An important consideration is that the baseline period (1985–2010) assumes that reefs are adapted to conditions of that particular time period, while in reality reef ecosystems have developed over centuries. In addition, the results presented here are based on the projections of a single earth system model. However, these projections are at least qualitatively useful in demonstrating the nature of the shifts in bioclimatic envelopes for coral reef ecosystems. 

The results describe how the spatial distribution of certain oceanographic environments strongly associated with reef development could shift while others experience less change. Continued investigation using a greater number of ensemble members and additional coupled climate models (as their results become available) are required to create models that are statistically more robust. Furthermore, these results do not consider the current ecological state of any coral-reef ecosystems, direct anthropogenic stressors such as pollution or fishing, the influence of invasive species, or molecular and ecological resilience of particular corals or coral-reef communities. 

In summary, this study suggests that the response of shallow tropical coral reefs to increased CO_2_ forcing is neither linear nor strictly latitudinal when considering a suite of representative variables known to influence coral ecology. Coral-reef ecosystems in different regions may respond in unique ways to the same forcing, and results suggest particular regions can be more suitable or less – an effect that remains consistent despite perturbations to model parameters. When individual basins are considered, conditions in which shallow-water coral-reef ecosystems presently exist in the Indian Ocean are shown to be most similar to future projections of global, tropical, physico-chemical ocean conditions. Coral reefs in this region may be most suited for persistence worldwide in future climate states. 

## Supporting Information

List S1 **Groups of highly correlated variables from principal components analysis.** Variable selected for subsequent analyses is indicated in bold. PAR = photosynthetically active radiation; CS = current speed; Ω_arag_ = aragonite saturation state.
(DOCX)

Table S1 **Correlation matrix of variables selected for species distribution model analysis.** Variables are listed along the top and left side, with ρ-correlations for each variable pair given in the table. CTS = cumulative thermal stress; PAR = photosynthetically active radiation; CS = current speed; Ω_arag_ = aragonite saturation state.
(DOCX)
