# Supplementary material for: Coral Reef Habitat Response to Climate Change Scenarios
Source: PLoS One. 2013 Dec 5;8(12):e82404. doi: 10.1371/journal.pone.0082404 (PMC3855618; doi:10.1371/journal.pone.0082404)
Supplement: List S1 — Groups of highly correlated variables from principal components analysis. Variable selected for subsequent analyses is indicated in bold. PAR = photosynthetically active radiation; CS = current speed; Ωarag = aragonite saturation state. (DOCX) [file pone.0082404.s001.docx]

**List S1:** Groups of highly correlated variables from principal components analysis. Variable selected for subsequent analyses is indicated in bold. PAR = photosynthetically active radiation; CS = current speed; Ω_arag_ = aragonite saturation state.

**Cumulative Thermal Stress**

PAR range

Ω_arag_ range

PO_4_ range

**PO_4_ maximum**

PO_4_ mean

PO_4_ minimum

**CS maximum**

CS minimum

CS mean

CS range

PAR maximum

Salinity range

**Salinity minimum**

Salinity maximum

Salinity mean

**Ω_arag_ minimum**

Ω_arag_ mean

Ω_arag_ maximum

PAR mean

**PAR minimum**

Temperature mean

Temperature minimum

Temperature maximum

Temperature range
