# Supplementary material for: Coral Reef Habitat Response to Climate Change Scenarios
Source: PLoS One. 2013 Dec 5;8(12):e82404. doi: 10.1371/journal.pone.0082404 (PMC3855618; doi:10.1371/journal.pone.0082404)
Supplement: Table S1 — Correlation matrix of variables selected for species distribution model analysis. Variables are listed along the top and left side, with ρ-correlations for each variable pair given in the table. CTS = cumulative thermal stress; PAR = photosynthetically active radiation; CS = current speed; Ωarag = aragonite saturation state. (DOCX) [file pone.0082404.s002.docx]

**Table S1:** Correlation matrix of variables selected for species distribution model analysis. Variables are listed along the top and left side, with ρ-correlations for each variable pair given in the table. CTS = cumulative thermal stress; PAR = photosynthetically active radiation; CS = current speed; Ω_arag_ = aragonite saturation state.

|  | **CTS** | **CS maximum** | **Ω_arag_ minimum** | **Salinity minimum** | **PAR minimum** | **PO_4_ maximum** |
| --- | --- | --- | --- | --- | --- | --- |
| **CTS** | 1 | -0.42 | -0.49 | 0.26 | -0.45 | 0.16 |
| **CS maximum** | -0.42 | 1 | 0.32 | -0.32 | 0.35 | 0.13 |
| **Ω_arag_ minimum** | -0.49 | 0.32 | 1 | 0.27 | 0.30 | -0.38 |
| **Salinity minimum** | 0.26 | -0.32 | 0.27 | 1 | -0.04 | -0.07 |
| **PAR minimum** | -0.45 | 0.35 | 0.30 | -0.04 | 1 | -0.12 |
| **PO_4_ maximum** | 0.16 | 0.13 | -0.38 | -0.07 | -0.12 | 1 |
